# The role of late reperfusion in ST-segment elevation myocardial infarction: a real-world retrospective cohort study

**DOI:** 10.1186/s12872-020-01479-0

**Published:** 2020-04-28

**Authors:** Qixin Guo, Jinyu Huang, Yong Shen, Guoxin Tong, Hong Li, Shasha Meng

**Affiliations:** 1 Nanjing Medical University, 818 East Tian Yuan Road, Jiang Ning District, Nanjing City, Jiangsu Province China; 2 Zhejiang University of Traditional Chinese Medicine, Hangzhou City, Zhejiang Province China; 3 Hangzhou First People’s Hospital, Hangzhou City, Zhejiang Province China

**Keywords:** ST-segment elevation myocardial infarction, Reperfusion therapy, Percutaneous Transluminal coronary intervention

## Abstract

**Background:**

Early reperfusion of the coronary artery has become the first choice for patients with ST-segment elevation myocardial infarction (STEMI). How to deal with patients who miss the time window for early reperfusion is still controversial. Based on real-world data, this study was conducted to explore whether percutaneous coronary intervention (PCI) has an advantage over standard drug therapy in patients who miss the optimal treatment window.

**Methods:**

Consecutive patients who were diagnosed with STEMI and met the inclusion criteria between 2009 and 2018 in our center were retrospectively included in this cohort study. The primary endpoint events were major adverse cardiac events (MACEs), including heart failure, sudden cardiac death, malignant arrhythmia, thrombi and bleeding events during the period of admission. Secondary endpoint events were components of MACEs. At the same time, we also evaluated angina pectoris at admission and discharge through Canadian Cardiovascular Society (CCS) grading.

**Results:**

This study enrolled 417 STEMI patients and divided them into four groups (PCI < 3 days, 14.87%; 3 days<PCI < 7 days, 21.104%; PCI > 7 days, 34.29%; MED, 29.74%). During the period of admission, MACEs occurred in 52 cases. The incidence of MACEs was 11.29, 7.95, 4.20 and 25.81% in the four respective groups (*p* < 0.0001). The MED group had higher rates of MACEs (OR = 3.074; 95% CI 0.1.116–8.469, *p* = 0.03) and cardiac death (OR = 3.027; 95% CI 1.121–8.169, *p* = 0.029) compared to the PCI group. Although both treatments were effective in improving CCS grade at discharge, the PCI group improved more significantly (*p* < 0.0001).

**Conclusions:**

In the real world, delayed PCI can be more effective in patients with angina symptoms at discharge and reduce the incidence of MACEs and cardiac death during hospitalization. The timing of intervention was independent of the occurrence of MACEs during hospitalization and of improvement in symptoms.

## Background

STEMI is on the rise in China, and the turning point in cardiovascular events has not occurred yet. Optimal treatment for STEMI includes early reperfusion or thrombolytic therapy. This is the cornerstone of contemporary treatment of STEMI, preventing myocardial necrosis and its consequences. Considering the current national conditions in China, it is difficult to carry out thrombolysis in primary hospitals [1–3]. After being transferred to the chest pain center [4], many patients miss the optimal PCI time or even refuse PCI. In contrast to developed countries [5, 6], approximately one-third of eligible patients receive primary PCI [7]. The rest are treated with either delayed PCI or conservative medication.

There is no definitive treatment strategy for STEMI patients who miss the optimal PCI window. Previous studies have shown that delaying PCI may be effective in maintaining cardiac function and improving cardiac remodeling in patients and can effectively alleviate electrophysiological disorders [8–11]. There have been some large randomized controlled trials (RCTs) that have challenged these ideas, and even though they have had similar experimental designs, they have had completely opposite results [12–16]. From the perspective of clinical analysis, their conclusions are very meaningful. The root cause is that RCT screening is so rigorous that the results apply only to a subset of the population. Therefore, these conclusions cannot be widely extended to clinical practice. Due to the sparse data from the real-world setting, the optimal management strategy for delayed patients with STEMI remains controversial. The aim of this study was to explore whether PCI has an advantage over standard drug therapy in a cohort of STEMI patients who miss the optimal treatment window based on real-world data from Hangzhou First People’s Hospital.

## Methods

### Study population

**Fig. 1:**
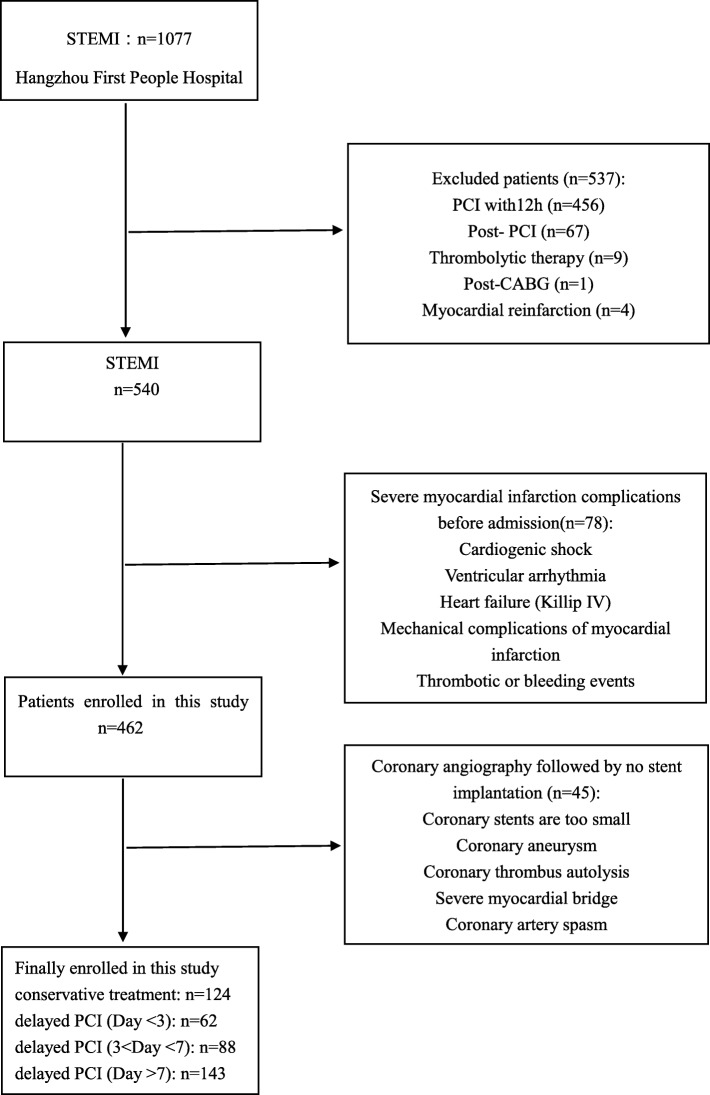
Flow chart of enrolment in this study

We retrospectively included all consecutive patients referred to Hangzhou First People’s Hospital for further treatment of myocardial infarction between 2009 and 2018. Treatment decisions were made by the physician and the patient in consultation, and the procedure and location of the stent placement was entirely up to the surgeon. Patients who met the inclusion criteria but did not meet the exclusion criteria were enrolled. The enrolled patients were divided into four subgroups based on predesigned criteria. In total, we included a total of 417 patients treated with PCI or conservative medications. A flow chart illustrating the patient selection process is presented in Fig. 1.

### Criteria for inclusion and exclusion

#### Inclusion criteria

- The patient was diagnosed with STEMI according to the STEMI guidelines in China [17]
- The onset of chest pain was greater than 12 h earlier

#### Exclusion criteria

- Previous PCI or coronary artery bypass graft (CABG) or thrombolytic therapy
- Myocardial reinfarction
- Severe myocardial infarction complications before admission
- Coronary angiography followed by no stent implantation

### Treatment

All patients were treated with optimal medications, including dual antiplatelet drugs (aspirin and clopidogrel), anticoagulants, angiotensin-converting enzyme inhibitors, β receptor blockers, and lipid-lowering therapy, for as long as the heart rate and blood pressure were not adversely affected, unless the use of these medications was clearly contraindicated. The clinician determined whether to give a dose of antiplatelet drugs by predicting whether the drug would reach an effective blood concentration at the time of surgery. Low-molecular-weight heparin (LMWH) was discontinued on the morning of PCI, common heparin was used intraoperatively to maintain the patient’s activated coagulation time (ACT) level, and LMWH was continued for 3–4 days after PCI. The use of additional instruments and drugs required during the procedure was entirely up to cardiovascular interventionists, such as thrombus aspiration catheters and β2/α3 receptor blockers. When myocardial infarction was complicated with multiple lesions, the culprit diseased vessels were treated preferentially, and non-culprit lesions were treated after 1 month.

### Data extraction

All blood biochemical results were obtained from the first blood samples taken within 24 h of admission. The evaluation of the coronary angiography results was performed entirely in the catheterization room by the surgeon and the first assistant. All CCS scores were assessed by the attending physician and recorded in the course of the disease. The extraction of the coronary angiography results and the CCS grades on admission and discharge were performed separately by the two authors (Qixin Guo and Yong Shen).

### Endpoint

The primary endpoint events were major adverse cardiac events (MACEs), including heart failure, sudden cardiac death, malignant arrhythmia, thrombus and bleeding events during the period of admission. Secondary endpoint events were components of major adverse cardiac events. We also evaluated angina pectoris at admission and discharge through CCS grading.

### Statistical analysis

Before the statistical operation, all the data were drawn into scatter graphs and tested for normality and homogeneity of variance. The main baseline characteristics of patients are described as frequencies for categorical variables and as mean ± standard deviation (SD) for continuous variables (normally distributed) or median with interquartile range (not normally distributed). The means of different groups were compared by one-way ANOVA (normality and independence) or Kruskal-Wails H rank sum test (independence but not normality). The comparison of multiple rates was performed using the common chi-square test. Pairwise comparisons between the groups were performed using either the chi-square test (ordered result variable) or the Mann-Whitney U test (disordered result variable) after correcting the *P* value.

**Fig. 2:**
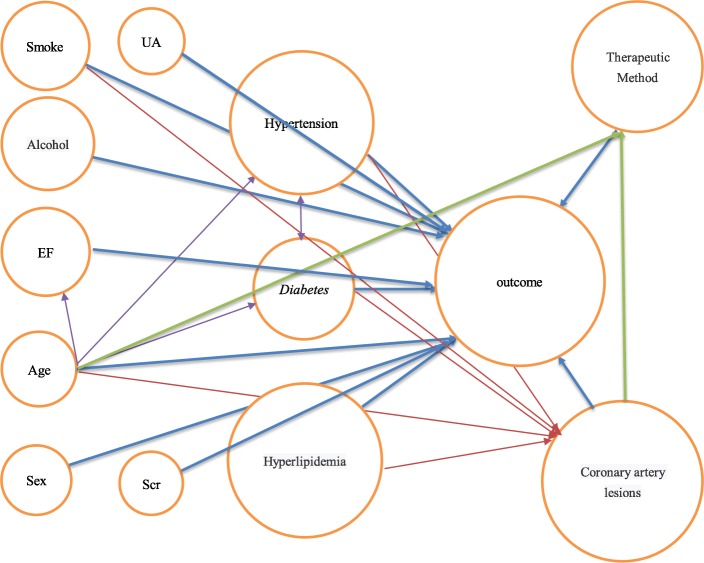
The DAG renderings are shown below, with the arrows representing cause and effect

Before we proceeded to multifactor logistic regression, we drew directed acyclic graphs (DAGs) [18, 19] to exclude possible mediating variables (Fig. 2). Then, we screened covariables through the effect change method and imported all possible covariables into the regression equation by using the enter method. The OR value of the drug treatment compared with PCI was recorded. Each variable was removed one by one, and a regression model was constructed to obtain the OR values of different treatment methods. We removed the variable that had the least effect on the OR value, and the OR value did not change by more than 10%. One by one, other variables were eliminated in the same way until all irrelevant variables were eliminated (Table 1). Finally, the selected covariates and control variables were combined to construct the regression model [20].

**Table 1:** Effect change method

variable	First round	second round	third round	fourth round	fifth round	sixth round	seventh round	eighth round	ninth round	tenth round
EF	3.328	3.379	3.402	3.398	3.379	3.27	3.169	3.218	3.256	3.581
LDL	2.876	2.871	2.871							
HDL	3.175	3.17	3.218	3.134	3.127	3.014	3.123	2.902		
UA	3.373	3.374	3.412	3.351	3.351	3.367	4.281	3.903	3.77	4.298
Scr	3.09	3.095	3.088	3.067	3.056	2.964				
Sex	2.846	2.849	2.851	2.846	2.804					
Hypertension	2.8	2.796	2.807	2.775	2.763	2.614	2.76			
Diabetes	2.895	2.897								
Smoking	2.859	2.854	2.856	2.863						
Alcohol	2.895									
Age	3.144	3.142	3.155	3.141	3.141	2.997	3.154	2.933	3.074	

*EF* ejection fraction, *LDL* Low density lipoprotein, *HDL* high-density lipoprotein, *SCR* serum creatinine, *UA* uric acid

The OR value in the range of 2.6037 to 3.1823 indicates that the change of OR value is less than 10%. After 10 rounds of screening, EF and UA are the variables that must be included in the regression model

All statistical analyses were conducted using SPSS software (version 23.0). A two-tailed *P*-value < 0.05 was considered statistically significant.

## Results

### Baseline characteristics

Table 2 summarizes baseline patient characteristics according to treatment modality and timing of intervention. The mean age was 71 years (58–79), and 68.3% of the patients were male. In the study, 58% had a medical history of hypertension, 2.6% had hyperlipidemia, and 20.9% had diabetes. Most of the patients in the MED group were elderly (compared with the other three groups, *P* < 0.0001). Significant differences between pairs of groups were also observed in CCS rank, UA, sex, and SCR.

**Table 2:** Baseline clinical and angiographic laboratory characteristics

	PCI < 3 days	3 days < PCI < 7 days	PCI > 7 days	MED	P	ALL PATIENTS
N	62 (14.87%)	88 (21.10%)	143 (34.29%)	124 (29.74%)		
Age	63 (51–72)	68 (57–77)	69 (58–78)	79 (69–84)	< 0.0001	71 (58–79)
Sex					0.03	
Male	46 (74.2%)	59 (67.0%)	107 (74.8%)	73 (58.9%)		68.30%
Female	16 (25.8%)	29 (33.0%)	36 (25.2%)	51 (41.1%)		31.70%
Hypertension	34 (54.8%)	51 (58%)	82 (57.3%)	75 (60.5%)	0.898	58.00%
Hyperlipidaemia	5 (8.1%)	1 (1.1%)	4 (2.8%)	1 (0.8%)	0.023	2.60%
Smoking	28 (45.2%)	38 (43.2%)	67 (46.9%)	47 (38.2%)	0.548	43.30%
Alcohol	17 (27.4%)	21 (23.9%)	30 (21.0%)	28 (22.6%)	0.786	23.00%
Diabetes	10 (16.1)	21 (23.9%)	37 (25.9%)	19 (15.3%)	0.122	20.90%
LDL	2.70 (2.09–3.58)	3.01 (2.26–4.72)	2.82 (2.07–3.81)	2.81 (2.14–3.88)	0.256	2.83 (2.11–3.94)
HDL	1.28 (1.00–1.70)	1.55 (1.03–2.47)	1.26 (0.91–2.28)	1.34 (1.01–2.35)	0.383	1.32 (0.99–2.33)
TC	4.79 (3.94–6.44)	5.45 (3.84–9.20)	5.15 (3.79–8.48)	4.99 (4.09–7.49)	0.288	4.99 (3.87–7.94)
UA	313 (249–413)	297 (231–361)	311 (239–401)	392 (264–507)	< 0.0001	324 (245–418)
Scr	86 (73–96)	80 (71–95)	86 (72–101)	98 (79–139)	< 0.0001	86 (74–107)
HbA1c	5.6 (5.2–7.1)	5.6 (5.2–6.9)	6.0 (5.5–7.3)	5.9 (5.5–6.6)	0.129	5.8 (5.4–6.9)
Glu	5.75 (4.84–7.08)	5.61 (4.77–6.50)	6.03 (5.10–7.28)	6.06 (4.89–7.87)	0.114	5.87 (4.94–7.44)
CCS						
Admission					< 0.0001	
I		3 (3.4%)	11 (7.7%)			3.40%
II	2 (3.2%)	20 (22.7%)	42 (39.4%)	8 (6.5%)		17.30%
III	9 (14.5%)	23 (26.1%)	35 (24.5%)	35 (28.2%)		24.50%
IV	51 (82.3%)	42 (47.7%)	55 (38.5%)	81 (65.3%)		54.90%
Discharge					< 0.0001	
I	20 (32.3%)	28 (31.8%)	53 (37.1%)	10 (8.1%)		26.60%
II	40 (64.5%)	59 (67.0%)	87 (60.8%)	66 (53.2%)		60.40%
III				14 (11.3%)		3.40%
IV	2 (3.2%)	1 (1.1%)	3 (2.1%)	34 (27.4%)		9.60%
LV Function						
EF	0.61 (0.52–0.67)	0.64 (0.53–0.70)	0.60 (0.51–0.66)	0.57 (0.51–0.67)	0.251	0.61 (0.52–0.67)
FS	0.32 ± 0.065	0.34 ± 0.084	0.312 ± 0.081	0.31 ± 0.090	0.215	0.32 ± 0.08
LVDd	4.99 (4.68–5.30)	4.99 (4.50–5.43)	5.12 (4.55–5.55)	5.05 (4.45–5.49)	0.635	5.03 (4.54–5.48)
LVDs	3.50 (3.10–4.21)	3.20 (2.78–3.82)	3.52 (2.95–4.23)	3.33 (2.86–4.06)	0.091	3.36 (2.95–4.12)
Angiographic						
LM	6 (9.7%)	4 (4.5%)	11 (7.7%)		0.459	7.20%
Single	24 (38.7%)	33 (37.5%)	54 (378%)		0.988	37.90%
More	36 (58.1%)	53 (60.2%)	88 (61.5%)		0.894	60.60%

*EF* ejection fraction, *LDL* low density lipoprotein, *HDL* high-density lipoprotein, *SCR* serum creatinine, *UA* uric acid, *Tc* cholesterol, *Glu* blood glucose, *LVDd* left ventricular end-diastolic dimension, *LVDs* left ventricular end-systolic dimension, *LM* left main coronary artery disease, *Single* single vessel lesion, *More* multiple vascular lesions, *CCS* Canadian cardiovascular society

### Primary endpoint

During hospitalization, 52 patients (12.47%) experienced a MACE: 26 (6.23%) had heart failure, 21 (5.04%) had cardiovascular death, 2 (0.48%) had myocardial reinfarction, 7 (1.68%) had malignant arrhythmia, and 6 (1.44%) had bleeding or thrombotic events (Table 3). The incidence of MACEs was 11.29, 7.95, 4.20 and 25.81% in the four respective groups (*p* < 0.0001). However, in the pairwise comparison results, no significant differences were found between the three PCI subgroups (Table 4), even after group merging. The MED group had higher rates of MACEs (OR = 3.074; 95% CI 0.1.116–8.469, *p* = 0.03) and cardiac death (OR = 3.027; 95% CI 1.121–8.169, *p* = 0.029) compared to the PCI group. Ejection fraction (EF), different treatment modalities and Uric acid (UA) were independent risk factors for MACEs in hospitals, while in-hospital deaths were only correlated with age and treatment modality. No statistically significant differences were found in the exploration results for other secondary end points (details can be found in Additional file 1).

**Table 3:** Primary and secondary outcomes

Event	PCI < 3 days (***n*** = 62)	3 days < PCI < 7 days (***n*** = 88)	PCI > 7 days (***n*** = 143)	MED (***n*** = 124)	P
MACE	7 (11.29)	7 (7.95)	6 (4.20)	32 (25.81)	< 0.0001
Heart failure	3	4	2	17	< 0.0001
Cardiac death	2	3	2	14	0.003
Recurrent MI	0	0	1	1	0.328
Malignant arrhythmia	2	0	0	5	0.009
Bleeding or thrombotic events	1	0	1	4	0.192

*MACE* major adverse cardiovascular events, *MI* myocardial infarction

The reason why the accumulative sum of secondary endpoint events is greater than that of primary endpoint events is that patients have accumulated a variety of adverse events during hospitalization

**Table 4:** Pairwise comparison of *P* values

CCS1	2	3	4
1	< 0.0001	< 0.0001	0.018
2		0.072	0.001
3			< 0.0001
CCS2			
1	0.901	0.468	< 0.0001
2		0.491	< 0.0001
3			< 0.0001
MACE			
1	0.491	0.056	0.022
2		0.23	0.001
3			< 0.0001
Heart failure			
1	0.933	0.143	0.066
2		0.145	0.028
3			< 0.0001
Cardiac death			
1	0.951	0.386	0.065
2		0.309	0.038
3			0.001

1 for PCI < 3 days group; 2 for 3 days < PCI < 7 days; 3 on behalf of the PCI > 7 days CCS1: CCS score at admission

*CCS2* CCS score at discharge, *MACE* major adverse cardiovascular events

The adjusted *P* value was 0.008333. The results were statistically significant only if the *P* value was less than 0.008333

### CCS classification score

**Fig. 3:**
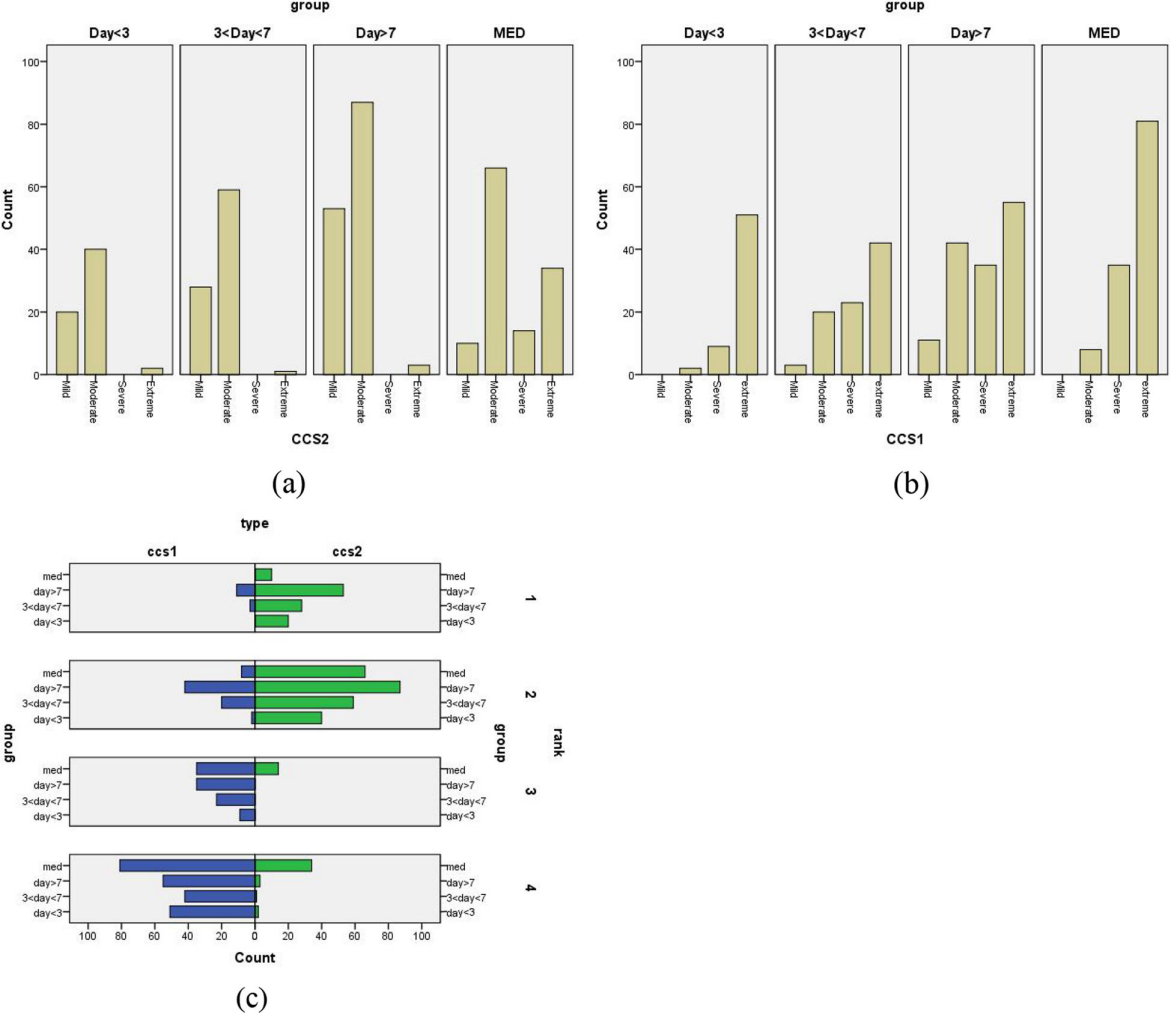
Comparison of discharge and admission CCS scores. CCS1 represents the CCS score at admission. CCS2 represents the CCS score at discharge. Mild Moderate Severe Extreme corresponds to CCS I II III IV respectively

There were significant differences in CCS classification at discharge and admission. When the differences between the groups were further studied, we found that there was no significant difference in CCS grade between groups 2 and 3 at admission, and the overall value was group 1 > group 4 > group 2 ≈ group 3 (Table 4). The same method was used to evaluate the CCS grade at discharge, and there was no statistically significant difference in CCS grade among the three PCI groups, with *P* values of 0.901, 0.468 and 0.491, respectively. The overall ranking was group 4 > group 1 ≈ group 2 ≈ group 3 (Table 4). Both conservative drug therapy and interventional therapy showed a significant decrease in CCS grading. In contrast, the interventional therapy was better alleviating patients’ subjective symptoms, but symptom alleviation had little relationship with the timing of intervention (Table 4, Fig. 3).

## Discussion

The principal findings of this real-world study of patients with STEMI who exceeded the optimal reperfusion window were as follows. 1. PCI treatment significantly reduced the incidence of MACEs and deaths in the hospital compared to optimal drug therapy. 2. PCI can significantly improve the patient’s symptoms after treatment and increase the satisfaction with the outcome of hospitalization.

This study focuses on areas that currently are not specifically recommended in the guidelines. Based on the conclusions of previous RCTs, this study can be a good supplement. The study group missed the optimal reperfusion window, and most of the patients still had significant angina symptoms when admitted to the hospital. The disease may deteriorate at any time. For these patients, the use of reperfusion therapy as soon as possible may be able to successfully protect the patient through the crisis period. The clinician’s focus on when reperfusion offers the greatest long-term benefit may actually increase the patient’s risk. It is theoretically and ethically impossible to include this population in RCTs. The results from observational studies have high intrinsic validity and can provide important references for clinical practice.

The current guidelines recommend direct PCI for STEMI patients with chest pain up to 12 h, with the indication extending up to 48 h in some patients [21, 22]. Residual anterograde coronary artery blood flow and reverse collateral circulation after myocardial infarction can ensure the survival of myocardial hibernating and myocardial suppressed cells, and saving these cells may prevent myocardial remodeling and electrophysiological disorders [21]. Such a pathophysiological basis may explain the appropriate relaxation of the treatment window for STEMI. The late open artery theory holds that the removal of vascular obstruction can improve the prognosis of patients. However, the results of a series of studies at OAT do not support this theory. The reasons for the different conclusions between the OAT study and other studies are as follows: 1. the baseline characteristics of the included populations are significantly different, and the time span of the population stratification is too large; 2. interventional devices and drugs have been updated; and 3. the patients enrolled in the OAT trial adhered to the ACC/AHA guidelines for the management of STEMI. Optimal drug regimens and careful management make the difference difficult to observe.

The detailed division of the definition of myocardial infarction contributed to the accuracy of the study. Recent research on non-ST-segment elevation acute coronary syndrome showed that the delayed group did not have an increased adverse prognosis compared to the early intervention group [22], but it was significantly better than conservative treatment [23]. Depending on the pathophysiology of different myocardial infarction types, the changes in nosocomial conditions and long-term prognosis are totally different [24]. Our study found that although PCI significantly improved the incidence of endpoint events, there was no difference between the time groups of different interventions, which is inconsistent with previous research conclusions [25, 26]. The combination of the PCI < 3 days group and the 3 days<PCI < 7 days group was compared with the PCI > 7 days group, and no significant results were obtained. This may be due to the limited number of cases and lack of long-term outcomes. In summary, based on the current evidence-based medical evidence, as an important influencing factor, PCI was performed within 3 days for patients whose condition might change in a short period of time and after 7 days for patients whose condition was relatively stable, which strongly correlated with the improvements of in-hospital events.

### Limitation

The present study has the following limitations: 1. this study is a single-center retrospective cohort study, the sample size is not large enough, and the exact results need to be supported by large-database studies. In addition, the retrospective nature of our study ensures that the results cannot be conclusive. 2. Many patients in our center were referred by local hospitals, which may result in deviations and partial data loss. For example, many patients in the group with PCI > 7 days were treated locally and transferred to our hospital for interventional surgery after stabilization. 3. During treatment, some patients were transferred to other hospitals for treatment or discharged automatically with unknown results. 4. Finally, we lost the follow-up records for our patients when we moved. Further research should focus on two areas. The first is to continue to follow up the patients and fill in the missing data. Survival analysis may yield more accurate results. Second, as cardiac intervention instruments and drugs are updated, large multicentric RCTs are also needed to guide the current treatment strategies.

## Conclusions

In the real world, our data suggested that delayed PCI can be more effective in patients with angina symptoms at discharge and reduce the incidence and MACEs and cardiac death during hospitalization. The timing of intervention was independent of the occurrence of MACEs during hospitalization and of the improvement in symptoms. We recommend further clinical trials to confirm this conclusion.

## Supplementary information

**Additional file 1.**

## Data Availability

All data that support the findings of this study are included in this published article [and its supplementary information files]. The datasets used and/or analysed during the current study are available from the corresponding author on reasonable request.

## Abbreviations

STEMI: ST-segment elevation myocardial infarction
PCI: Percutaneous coronary intervention
MACEs: Major adverse cardiac events
CCS: Canadian cardiovascular society
RCTs: Randomized controlled trials
CABG: Coronary artery bypass graft
LMWH: Low-molecular-weight heparin
ACT: Activated coagulation time
SD: Standard deviation
DAG: Directed acyclic graphs
EF: Ejection fraction
UA: Uric acid
