# Supplementary material for: The role of late reperfusion in ST-segment elevation myocardial infarction: a real-world retrospective cohort study
Source: BMC Cardiovasc Disord. 2020 Apr 28;20:207. doi: 10.1186/s12872-020-01479-0 (PMC7189551; doi:10.1186/s12872-020-01479-0)

Table 1 Chi-square test for malignant arrhythmia

Table 2 Chi-square check for MACE events

Table 3 Chi-square test of death event

Table 4 Chi-square test for heart failure events

Table 5 Chi-square test for thrombus and bleeding events

Table 6 Chi-square test for secondary myocardial infarction

Table 7 Logistic regression results of MACE events

Table 8 Logistic regression of malignant arrhythmia

Table 9 Logistic regression results of death events

Table 10 Logistic regression of heart failure

Table 11 Logistic regression of thrombus and bleeding events

Table 12 Combined comparison chi-square test


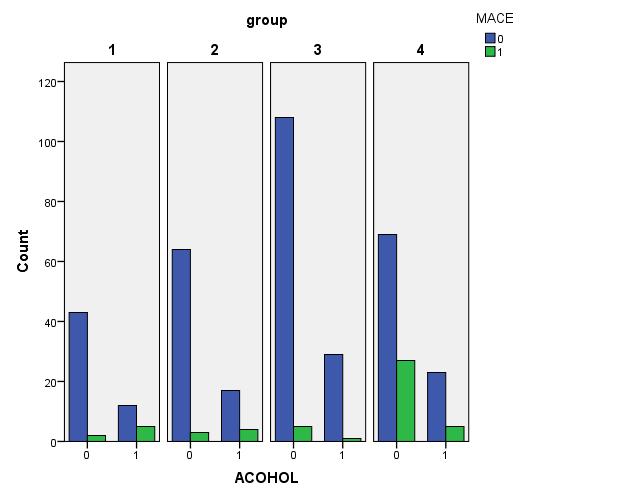

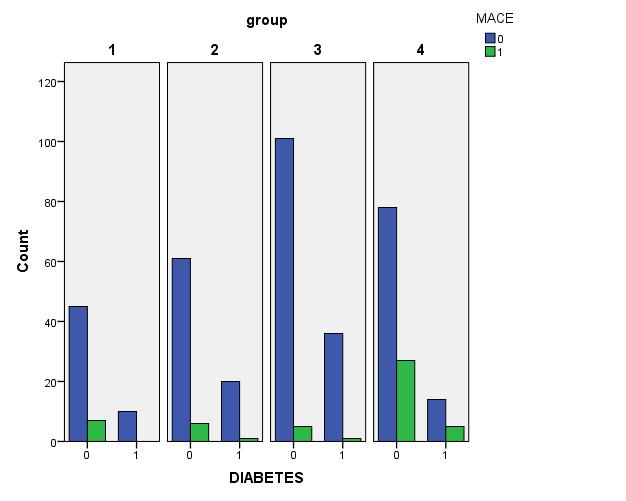

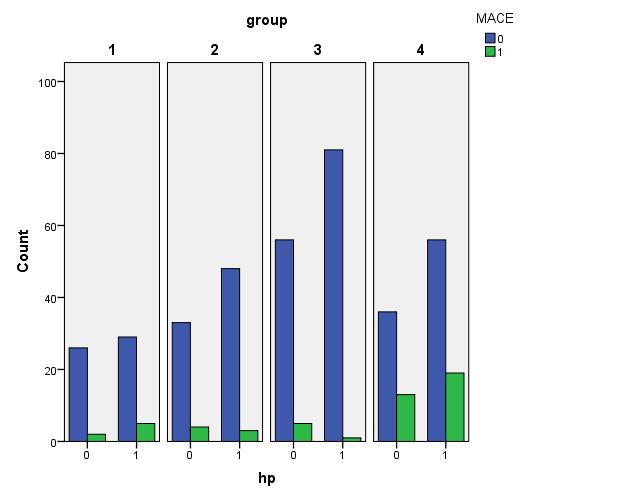

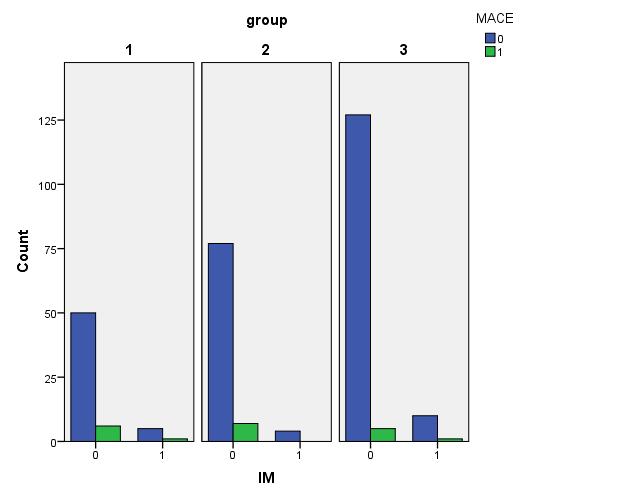

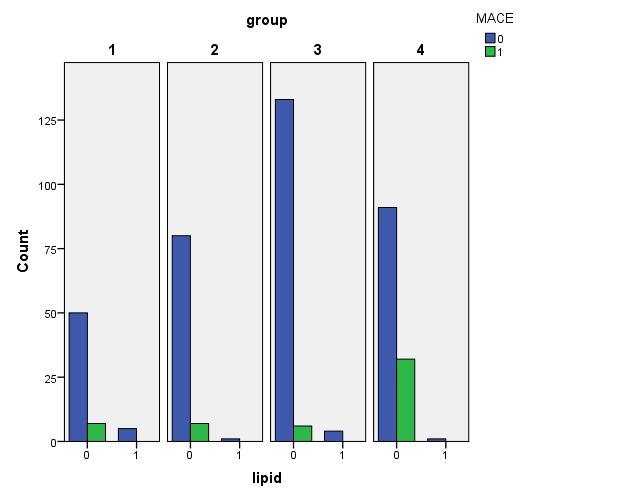

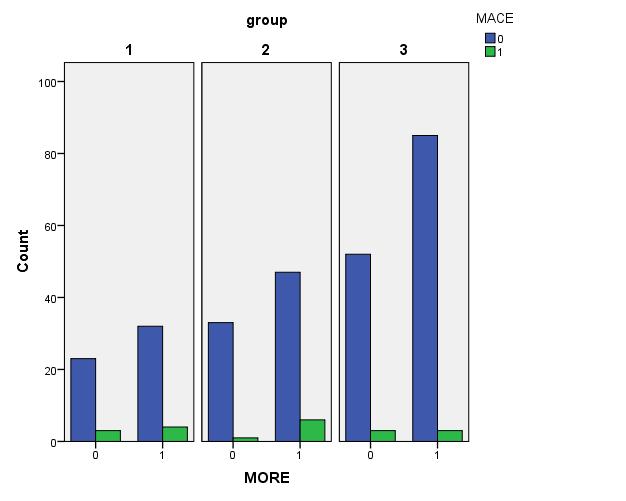

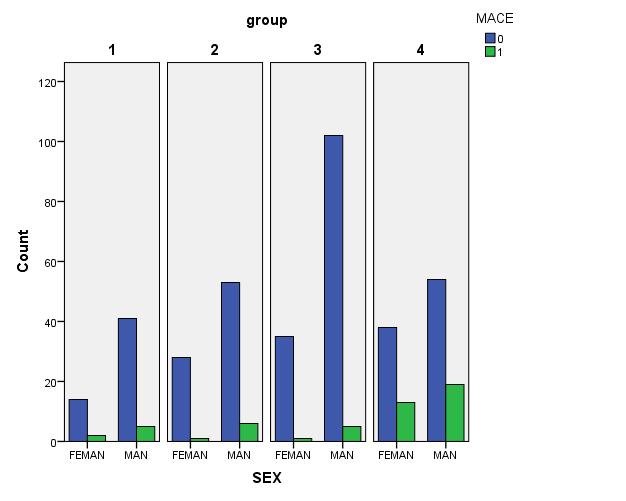

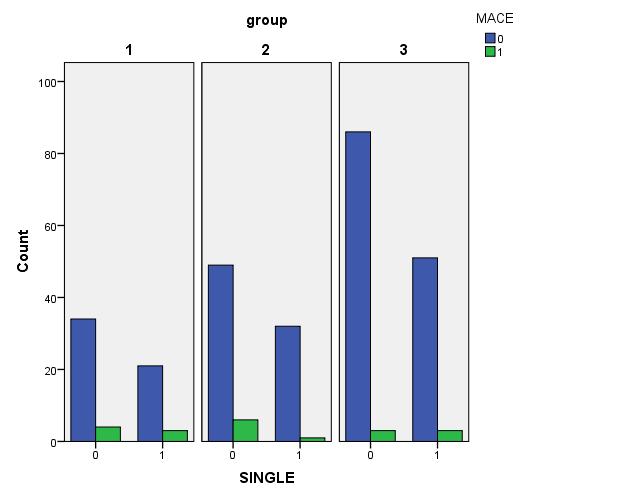

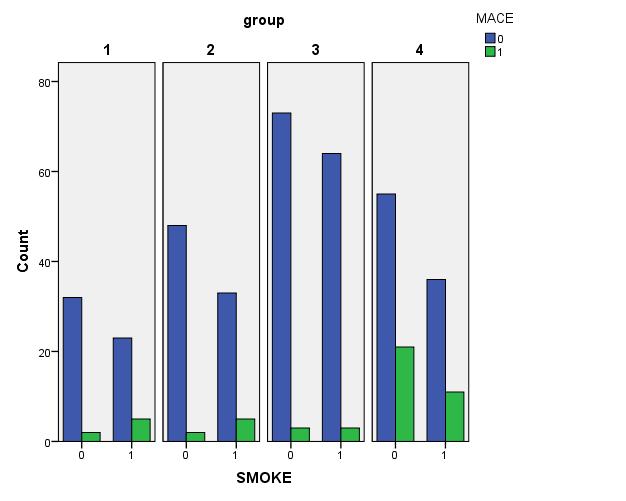

Supplement: Supplementary file 1 — Additional file 1. [file 12872_2020_1479_MOESM1_ESM.docx]
